# Adolescents with full or subthreshold anorexia nervosa in a naturalistic sample – characteristics and treatment outcome

**DOI:** 10.1186/s40337-017-0135-5

**Published:** 2017-03-02

**Authors:** Katarina Lindstedt, Lars Kjellin, Sanna Aila Gustafsson

**Affiliations:** University Health Care Research Center, Faculty of Medicine and Health, Örebro University, SE-701 82 Örebro, Sweden

**Keywords:** Adolescents, Anorexia nervosa, Eating disorders, Naturalistic sample, Treatment

## Abstract

**Background:**

Anorexia Nervosa (AN) destroys developmentally important early years of many young people and knowledge is insufficient regarding course, treatment outcome and prognosis. Only a few naturalistic studies have been conducted within the field of eating disorder (ED) research. In this naturalistic study we included adolescents with AN or subthreshold AN treated in outpatient care, and the overall aim was to examine sample characteristics and treatment outcome. Additional aims were to examine potential factors associated with remission as an outcome variable, and possible differences between three time periods for treatment onset.

**Methods:**

Participants were identified through the Swedish national quality register for eating disorder treatment (SwEat), in which patients are registered at treatment onset and followed up once a year until end of treatment (EOT). Inclusion criteria were: medical or self-referral to one of the participating treatment units between 1999 and 2014, 13–19 years of age at initial entry into SwEat and diagnosed with AN or subthreshold AN. The total sample consisted of 3997 patient from 83 different treatment units.

**Results:**

The results show that 55% of the participants were in remission and approximately 85% were within a healthy weight range at EOT. Of those who ended treatment according to plan, 70% were in remission and 90% within a healthy weight range. The average treatment duration was approximately 15 months. About one third of the patients terminated treatment prematurely, which was associated with a decreased chance of achieving remission. Remission rates and weight recovery increased over time, while treatment duration decreased. Considering treatment outcome, the results did not show any differences between patients with AN or subthreshold AN.

**Conclusions:**

The present study shows a relatively good prognosis for adolescent patients with AN or subthreshold AN in routine care and the results indicate that treatment for adolescents with ED in Sweden has become more effective over the past 15 years. The results of the present study contribute to the scope of treatment research and the large-scale naturalistic setting secures the generalizability to a clinical environment. However, more research is needed into different forms of evidence, new research strategies and diversity of treatment approaches.

**Trial registration:**

Registered in FOU in Sweden (Researchweb.org) 2014-04-14, ID nr 147301.

## Plain english summary

This study is one of few studies within the field of eating disorder (ED) research that is conducted in a natural treatment setting. In this study we included adolescents with Anorexia Nervosa (AN) or comparable symptoms treated in outpatient care, and the overall aim was to examine the characteristics of the sample and treatment results. Additional aims were to examine possible treatment factors that could be associated with being free from an ED diagnosis at end of treatment (EOT), and possible differences between three time periods for start of treatment. Participants were identified through the Swedish national quality register for eating disorder treatment (SwEat) and a total of 3997 patients from 83 different treatment units were included. The results show that 55% of the participants were free from an ED diagnosis at EOT and about 85% were within a healthy weight range. The results show no differences between patients with AN or with comparable symptoms, but terminating treatment prematurely imply a decreased chance of achieving remission. The results indicate that treatment has become more effective over the past 15 years.

## Background

Anorexia Nervosa (AN) is a severe form of eating disorder (ED) that is costly, both in terms of personal suffering and health economy on an individual and societal level, and it destroys developmentally important early years for many adolescent girls and boys [1, 2]. Although people of all ages are affected, AN often has its onset during adolescence and mainly affects girls between 15 and 19 years [3–5]. Despite recent advances within the ED research field, there is still inadequate knowledge about the course, treatment outcome and prognosis of adolescent AN [6]. Early treatment interventions have been shown to be important for the best effects [1, 7, 8]. When treatment is delayed, the risk increases for more severe and prolonged symptoms [2, 9]. However, the treatment often progresses slowly [10] and is characterized by high dropout rates [11]. According to previous studies, this is due to comorbidity with other psychiatric diagnoses [1, 7, 12], difficulties in responding to therapy when in starvation [13], patients’ denial of their problems [1, 7] and unwillingness to gain weight [14]. It has also been suggested that the physical and cognitive development that occurs during adolescence, in addition to major life changes like moving away from home, make treatment planning complex [1, 2]. Approximately 20–40% relapse within the first year after end of treatment (EOT) [15, 16], a rate that is somewhat lower among adolescents than among adults [16]. Complete recovery is expected in about 50% of AN cases [8, 17, 18].

Only a few naturalistic studies have been conducted within the field of adolescent ED, (e.g. [19, 20]). Naturalistic studies can add valuable knowledge about the impact of experimental results and outcome of various treatments in real life settings, and about descriptive baseline data for patients. In this naturalistic study we included adolescents with AN or subthreshold AN, according to the Diagnostic and Statistical Manual of Mental Disorders IV (DSM-IV) [21]. We based this on results from previous studies, showing that patients with subthreshold AN most often suffer from symptoms to the same extent as patients with AN, despite a higher BMI (Body Mass Index, kg/m^2^) in general [22–24]. This is also in line with the updated criteria in Diagnostic and Statistical Manual of Mental Disorders 5 (DSM-5) [25], in which the definition of AN has been broadened.

The overall aim of this study was to examine sample characteristics and treatment outcome in a naturalistic sample of adolescents with AN or subthreshold AN, treated in outpatient care. Additional aims were to examine potential factors associated with remission as an outcome variable, and possible differences in sample characteristics and treatment outcome between three time periods for treatment onset.

## Methods

Participants in the present study were identified through the Swedish national quality register for eating disorder treatment (SwEat). SwEat is a longitudinal internet-based quality assurance register, developed in 1999, that includes all specialist ED units in Sweden and a fair number of general psychiatric units. A total of 108 units participated in SwEat between 1999 and 2014. The objectives of SwEat is to document clinically important key variables, such as waiting time, treatment duration, different types of treatment interventions (e.g. outpatient, day patient or inpatient treatment) and treatment outcome [26]. Information is registered in SwEat when it is established that the patient has an ED diagnosis, the unit intends to treat the patient and the patient has given her/his consent to registration. The patient is initially registered in SwEat at treatment onset and then followed up once a year until EOT. Each patient might be initially registered more than once, since a patient is initially registered again if terminating treatment and later on entering a new treatment episode. SwEat includes data from patients of all ages and both genders. A total of 17611 initial registrations were made in SwEat between 1999 and 2014, when there was a change in methodology and the original version of SwEat was revised.

### Study sample

**Fig. 1:**
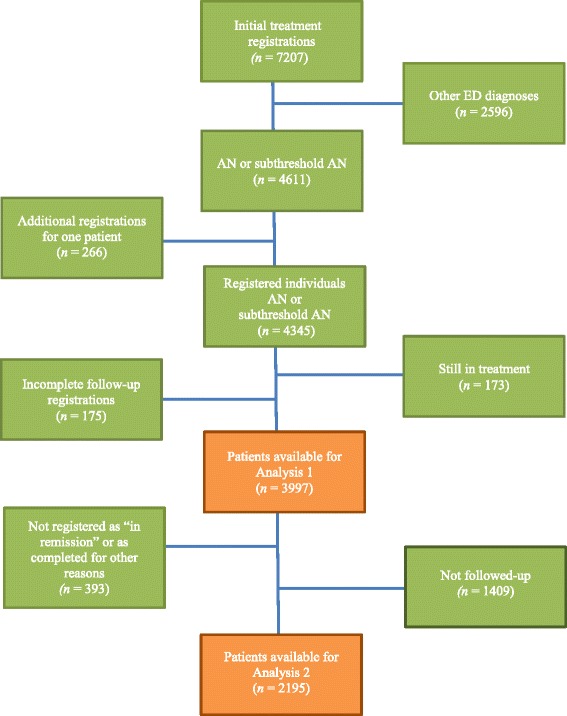
Flowchart, SwEat registrations of patients 13–19 years during 1999–2014

All patients who met the following criteria were included in the study: medical or self-referral to one of the participating treatment units between 1999 and 2014, 13–19 years of age at initial entry into SwEat and diagnosed with AN or subthreshold AN according to DSM-IV [21], which during the years examined constituted the basis for diagnoses at Swedish ED units (see Fig. 1 for a flow chart). The patients were diagnosed by experienced staff in consultation with multidisciplinary teams, and at most units on the basis of a structured interview guide. Since 2008 the Structured Eating Disorder Interview (SEDI) has been used at Swedish ED units [27] and before that the most commonly used interview guide was the Rating of Anorexia and Bulimia interview (RAB) [28]. In the present study, we focused on individuals instead of treatments and therefore included solely information about the first treatment episode for patients who had more than one episode registered. By choosing the first treatment episode, we included mainly information about patients who entered treatment for the first time. Excluded were patients who i) were followed up but had an incomplete follow-up registration, due for instance to inaccurate data, or ii) were still in treatment when the data collection was discontinued in 2014.

In the first analysis, when examining sample characteristics at treatment onset, all patients remaining considering the mentioned criteria above were included. In the second and third analysis we included only patients who were registered at EOT as “in remission” or as completed for other reasons. In the second analysis, these patients were divided into three groups based on different time periods for treatment onset; Period 1 (1999–2004), Period 2 (2005–2009) and Period 3 (2010–2014). A total of 83 treatment units were represented in the study, of which 42 were specialist ED units. Patients in the present study received inpatient, day patient and/or outpatient treatment, such as individual psychotherapy, family therapy and group interventions.

Of the patients in the total sample (*n* = 3997), 35% were lost to follow-up in SwEat. Comparisons between followed up and non-followed up patients regarding baseline characteristics showed only a few differences between the two groups, of which the most obvious was that followed-up patients had more social complications at treatment onset (15.6% vs 9.9%, *p* = <.001). Furthermore, the patients lost to follow-up had been ill for rather longer when entering treatment (2.2 years vs 1.9 years, *p* = <.001) and were younger at first symptoms (14.4 years vs 14.7 years, *p* = <.001).

### Measures

SwEat requires information about the following variables used in the present study (Table 1).

**Table 1:** Data collected at SwEat registration^1^

	Initial registration	Follow-up registration
Does the patient have symptoms consistent with a specified or unspecified ED, according to DSM-IV? (Yes/No)	X	
Does the clinic intend to treat the patient? (Yes/No)	X	
Has the patient been informed about SwEat and given his/her oral consent for registration? (Yes/No)	X	
Civic registration number (YYYY-MM-DD-XXXX, the last four digits comprise the Swedish social security number and specify gender)	X	X
Date of treatment onset (YYYY-MM-DD)	X	
The patient’s current ED diagnosis (DSM-IV Axis I/No current ED)	X	X
The patient’s age at onset of ED symptoms (years)	X	
The patient’s current weight (kg, to one decimal)	X	X
The patient’s current height (cm, to one decimal)	X	X
Are there one or several factors that clearly complicate treatment? (Yes, of psychiatric nature/Yes, of somatic nature/Yes, of social nature/No)	X	
Who referred the patient to the unit? (Patient/Relative/Other treatment unit or school)	X	
What previous contact with the health care services did the patient have for the eating disorder? (This is the first contact/Previous contact of an occasional nature/Previous treatment)	X	
Is the patient living alone or with others? (Single/With children/With parents/With partner/Other)	X	X
The patient’s employment (Studying/Working/On sick leave)	X	X
Is the treatment finished? (Yes/No)		X
If the treatment is finished: What date? (YY-MM-DD)		X
If the treatment is finished: How did it end? (In agreement between patient and therapist/Patient terminated treatment prematurely/Patient was referred to another treatment unit/Other reason)		X

^1^This table only includes data presented in the study. The SwEat registration contains additional data

The registration form contains boxes for each response alternative, and the system requires that all boxes are ticked before the form can be submitted. Even so, most of the variables have 1–3% missing or invalid answers.

In the present study the following variables were selected as outcome measures:

#### Remission

Patients not fulfilling criteria for any ED diagnosis at follow-up were categorized as being in remission.

#### Weight status

Height and weight, either measured by a therapist or self-reported by the patient, were used to calculate the patients’ BMI at initial registration and follow-up. Based on the BMI percentile method for calculating expected body weight, we assessed the patients as being within a normal or low weight range. This was done in accordance to a previous study describing and recommending this method [29] and by using Swedish reference values for BMI, adjusted for age and gender [30].

#### Premature termination of treatment

The term is used for patients who do not complete treatment, regardless of reason [31]. In the present study, since there were no prescribed treatment doses and the length of treatment was not determined at treatment onset, the term was used to categorize patients terminating treatment either on their own or their parents’ initiative or due to referral to another treatment unit.

#### Treatment duration

Treatment duration was measured in months and possible to calculate for the patients who had a registered date for their first and last treatment session (*n* = 1904).

#### Sick leave

At both initial registration and follow-up, information on employment is requested and clinicians are asked to specify if the patient is on sick leave from work or school. In the present study, we did not differentiate between patients on full or part-time sick leave.

### Statistical analyses

Statistical analyses were carried out using IBM SPSS Statistics 22. In order to compare variable values between two different patient groups (e.g. followed up and non-followed up patients) we used Pearson’s chi-square test and independent samples *t*-test. When exploring possible differences between three time periods we used Pearson’s Chi-square test and one-way ANOVA, and analyzed post hoc by examining possible differences between two groups at one time and by using Scheffe’s post hoc test. Finally, we conducted logistic and multiple logistic regressions in order to examine factors associated with remission as an outcome variable. The regressions were performed using only those independent variables found to differ significantly between the groups *In remission* and *Not in remission*. In order to correct for multiple analyses, we used Bonferroni correction with thresholds set at *p* = <.001 throughout the study.

## Results

Most patients were adolescent girls who, at the time of treatment onset, were studying and living at home with their parents or other relatives (Table 2). Approximately 60% were considered to have a low weight when entering treatment and almost as many had an AN diagnosis. One third of the patients had previous experiences of treatment for ED and complicating social, psychiatric or somatic factors were registered in more than one third of the cases, of which most were psychiatric. In a few cases patients were registered as being on sick leave. Approximately 60% of the patients had been referred to treatment by for instance another treatment unit or a school health service. On average the patients had been ill for two years when entering treatment. Including only those entering treatment for the first time (*n* = 2737) illness duration was approximately 1.7 years (min = 0, max = 11.9; SD = 1.6).

**Table 2:** Total sample characteristics at treatment onset

	Total sample (*n* = 3997)
Girls (%)	3823 (95.6)
Studying (%)	3574 (89.4)
Living with parents/other relatives (%)	3785 (94.7)
Low weight (%)	2385 (59.7)
AN (%)	2284 (57.1)
Previous ED treatment (%)	1221 (30.5)
Social complications (%)	543 (13.6)
Psychiatric complications (%)	685 (17.1)
Somatic complications (%)	149 (3.7)
Sick leave (%)	265 (6.6)
Referred to treatment by other treatment unit or school (%)	2424 (60.6)
Age at first symptoms of ED M (SD)	14.6 (1.9)
Age when entering treatment M (SD)	16.6 (1.8)
Illness duration at treatment onset (in years) M (SD)	2.0 (1.8)

*M* mean, *SD* standard deviation

Separate analyses of patients who were registered at EOT as “in remission” or as completed for other reasons, showed that just over two thirds ended treatment according to an initial treatment plan or because they were in remission. Other patients ended treatment prematurely, either on their own or their parents’ initiative (*n* = 283, 12.9%) or due to referral to another treatment unit (*n* = 350, 15.9%). The average treatment duration was approximately 15 months (min = 1, max = 135). Just above 55% of the patients were in remission at EOT and 16% of the patients were still considered to have a low weight (Table 3). Separate analyses of patients who ended according to plan (*n* = 1564) revealed that just under 70% were in remission at EOT and approximately 10% were considered to have a low weight.

**Table 3:** Treatment characteristics and treatment outcome among patients with completed treatments; results for total sample and comparisons between three time periods

	Completed treatments (*n* = 2195)	Time period			Sign.	Post hoc	
		1 1999–2004 (*n* = 457)	2 2005–2009 (*n* = 1219)	3 2010–2014 (*n* = 519)			
Baseline characteristics							
Age at first ED symptoms M (SD)	14.7 (1.9)	14.6 (1.9)	14.8 (1.8)	14.5 (1.9)	.007	1-2	.250
						2–3	.007
						1–3	.499
Age at treatment onset M (SD)	16.6 (1.8)	16.5 (1.9)	16.6 (1.8)	16.4 (1.8)	.071	1-2	.644
						2–3	.058
						1–3	.511
Referred to treatment by other treatment unit or school (%)	1374 (62.6)	300 (65.6)	771 (63.2)	303 (58.4)	.050	1-2	.363
						2–3	.056
						1–3	.020
Previous ED treatment (%)	667 (30.4)	199 (43.5)	355 (29.1)	113 (21.8)	<.001	*1-2*	*<.001*
						2–3	.002
						*1*–*3*	*<.001*
Social complications (%)	355 (16.2)	103 (22.6)	183 (15.0)	69 (13.3)	<.001	*1-2*	*<.001*
						2–3	.352
						*1*–*3*	*<.001*
Psychiatric complications (%)	362 (16.5)	104 (22.9)	189 (15.5)	69 (13.3)	<.001	*1-2*	*<.001*
						2–3	.236
						*1*–*3*	*<.001*
Somatic complications (%)	88 (4.0)	23 (5.1)	45 (3.7)	20 (3.9)	.440	1-2	.209
						2–3	.871
						1–3	.362
AN (%)	1240 (56.5)	310 (67.8)	683 (56.0)	247 (47.6)	<.001	*1-2*	*<.001*
						2–3	.001
						*1*–*3*	*<.001*
Low weight (%)	1231 (56.1)	281 (61.5)	697 (57.2)	253 (48.7)	<.001	1-2	.111
						2–3	.001
						*1*–*3*	*<.001*
Sick leave (%)	137 (6.2)	44 (9.6)	74 (6.1)	19 (3.7)	.001	1-2	.011
						2–3	.041
						*1*–*3*	*<.001*
Outcome variables							
Remission (%)	1220 (55.6)	221 (48.4)	682 (55.9)	317 (61.1)	<.001	1-2	.006
						2–3	.048
						*1*–*3*	*<.001*
Low weight (%)	358 (16.3)	97 (21.3)	196 (16.1)	65 (12.5)	.001	1-2	.012
						2–3	.057
						*1*–*3*	*<.001*
Premature termination of treatment (%)	633 (28.8)	127 (27.8)	357 (29.3)	149 (28.7)	.832	1-2	.547
						2–3	.808
						1–3	.750
Treatment duration (months) M (SD)*	14.8 (11.8)	19.2 (15.8)	14.4 (10.8)	11.4 (7.7)	<.001	*1-2*	*<.001*
						*2*–*3*	*<.001*
						*1*–*3*	*<.001*
Sick leave (%)	72 (3.3)	22 (4.8)	44 (3.6)	6 (1.2)	.004	1-2	.248
						2–3	.005
						1–3	.001

*(*n* = 1904)

*M* mean, *SD* standard deviation

The number of patients who were considered to have a low weight at treatment onset were lower in period 3 and for each period relatively fewer patients had been entering treatment with an AN diagnosis. The proportion of patients entering treatment with experiences of previous ED treatment and social or psychiatric complications were lower in period 2 and 3. Examination of treatment outcome revealed that treatment duration had shortened for each time period. There were also a reduced number of patients who were considered to have a low weight at EOT in period 3, and a larger number of patients in remission.

Patients who terminated treatment prematurely had a decreased chance of achieving remission (Table 4). Remission was more likely for patients who entered treatment in time period 3 compared to period 1. Also, although not significant at < .001, there was a clear tendency in the adjusted analyses that longer treatment duration was positively correlated to remission.

**Table 4:** Logistic and multiple logistic regression analyses with remission as an outcome variable

		In remission (*n* = 1220)	Not in remission (*n* = 975)	Unadjusted		Adjusted	
				β (95% CI)	Sign.	β (95% CI)	Sign.
Previous ED treatment (%)	Yes	363 (29.8)	304 (31.2)	0.94 (0.78 1.12)	.471	1.02 (0.81 1.28)	.873
	No	857 (70.2)	671 (68.8)	Ref			
Psychiatric complications (onset) (%)	Yes	174 (14.3)	188 (19.3)	0.70 (0.56 0.88)	.002	0.84 (0.63 1.10)	.204
	No	1043 (85.7)	787 (80.7)	Ref.			
AN (onset) (%)	Yes	688 (56.4)	552 (56.6)	0.99 (0.84 1.17)	.917	1.05 (0.82 1.34)	.727
	No	532 (43.6)	423 (43.4)	Ref.			
Low weight (onset) (%)	Yes	670 (54.9)	561 (57.5)	0.90 (0.76 1.07)	.219	0.79 (0.62 1.00)	.054
	No	550 (45.1)	414 (42.5)	Ref.			
Treatment duration (months) M (SD)		15.4 (10.9)	14.1 (12.6)	1.01 (1.00 1.02)	.013	1.02 (1.01 1.02)	.001
Premature termination of treatment (%)	Yes	128 (10.5)	503 (51.6)	0.11 (0.09 0.14)	<.001	0.15 (0.12 0.19)	*<.001*
	No	1092 (89.5)	472 (48.4)	Ref.			
Time periods (%)	1 1999-2004	221 (18.1)	236 (24.2)	0.60 (0.46 0.77)	<.001	0.51 (0.37 0.70)	*<.001*
	2 2005-2009	682 (55.9)	537 (55.1)	0.81 (0.66 1.00)	.048	0.87 (0.68 1.12)	.279
	3 2010-2014	317 (26.0)	202 (20.7)	Ref.			

*M* mean, *SD* standard deviation

## Discussion

This study is one of few that investigates treatment outcome in a naturalistic setting for adolescents with full or subthreshold AN. The main results show that 55% of the participants were rated by clinicians as being in remission and approximately 85% were within a healthy weight range at EOT. These results are in line with other naturalistic studies examining treatment outcome among adolescents with AN (e.g. [19, 20, 32]). However, some of these studies are old and/or differ from our study in elementary aspects, including assessment intervals and outcome estimates. It has been suggested that the definition of recovery used in a study has an important impact on outcome estimates [33]. In the present study, we refrained from defining recovery and confined ourselves to only use the term “in remission” for patients not fulfilling criteria for any ED diagnosis. It is not possible either to make a full comparison of our results with randomized controlled trials within this field (e.g. [15, 34–36]), due for instance to divergent study structures and different ways of measuring outcome.

Approximately 70% of those who ended treatment according to plan were rated as being in remission and 90% were within a healthy weight range. This indicates the importance of completing treatment, which will be discussed later on.

The results in the present study also show that remission rates and weight recovery increased over time, while treatment duration decreased. The fact that patients over the years became healthier when entering treatment is a possible explanation. However, this cannot fully explain these results since neither low weight nor complicating factors at treatment onset was associated to a poor outcome. The results might therefore indicate that treatment has become more effective over the past 15 years. This seems promising, but needs to be studied further since there is not enough knowledge about causal factors and the generalizability of such a trend.

The large number of patients within a healthy weight range is, needless to say, a positive result. However, when studying adolescents it is important to bear in mind that a categorization in low and normal weight based on BMI is difficult. Despite a BMI within a seemingly normal range, a young patient may have a low weight or even be underweight in relation to his or her own weight curve. This information is unfortunately hard to capture within SwEat, because of the large number of patients. A normal weight does not necessarily mean that the patient is healthy or recovered, and suffering from AN can be critical regardless of weight [2]. However, it is suggested in previous studies that BMI is an important prognostic factor [37] and that significant weight gain at EOT is a reliable predictor of recovery in adolescents with AN [38, 39].

Almost 60% of the adolescents had an AN diagnosis at treatment onset, which in comparison to what is presented in previous studies is a fairly large proportion [22, 40]. In these studies it is suggested that the majority of adolescents seeking ED treatment have variants of subthreshold diagnoses. Considering treatment outcome, the results in our study did not show any differences between patients with AN or subthreshold AN. In fact, the number of patients in remission did not differ at all between the two groups. This corresponds to previous results suggesting that patients with AN or subthreshold AN in general suffer from symptoms to the same extent [22–24], but runs counter to another study suggesting that recovery is eight times more likely among patients with subthreshold AN [41].

Patients in time period 2 and 3 were more often considered to have a normal weight when entering treatment, were less often diagnosed with AN, had fewer experiences of previous ED treatment and less social and psychiatric complicating factors. However, there was no difference in illness duration at treatment onset between the different time periods, as one could expect. Instead, adolescents during later years might have been seeking treatment for less serious conditions, perhaps due to easier access to health care and increased awareness of ED in society.

In our study, the average age when entering treatment was 16.6 years, which is in accordance to results from a British study suggesting that the peak age of presentation for treatment is 15–19 years [5]. Age at first symptoms of illness was on average 14.6 years, which indicates approximately two years of illness duration at treatment onset. When excluding patients with experiences of previous ED treatment, illness duration at treatment onset was slightly shorter. In previous studies it is suggested that duration between onset of illness and initiation of treatment is often rather long, in particular when it comes to those with an early onset of illness [7, 9]. This may partly reflect the fact that many people with AN do not see their symptoms as problematic but more as a part of their identity and that they lack internal motivation to recover [1, 7]. Many adolescents are likely to have atypical presentations of ED, which increases the risk for delayed diagnoses and significant complications [1, 2, 42, 43]. Approximately one third of the patients in the present study terminated treatment prematurely, either on their own or their parents’ initiative or due to referral to another treatment unit. This corresponds to results from previous studies, suggesting a proportion of 20–40% [11]. Premature termination of treatment is considered a problem within several psychiatric disorders and in particular within the field of ED and AN [44, 45]. For example, as this study also showed, terminating treatment prematurely reduces the chance of achieving remission while completing treatment increases the chances of a good outcome [11, 35]. As mentioned earlier, as many as 70% of those who ended treatment according to plan were in remission and 90% within a healthy weight range. Which clinical characteristics and factors that can be associated with premature termination have yet to be discovered, but one suggestion is discrepancy between patient preferences and expectations about treatment that may account for non-adherence [1]. It may also be linked with treatment dissatisfaction, which will be explored in an upcoming study based on data from SwEat. In the present study, treatment duration was approximately 15 months and it has been suggested that treatment should last at least six months for a desirable outcome [7, 20].

The fact that complicating social, psychiatric or somatic factors were registered in more than one third of the cases, of which most were psychiatric, is not surprising. Although only a few studies have looked at social or somatic factors (e.g. [46]), psychiatric comorbidity is well known to be a complicating factor for these patients [4, 12, 41, 46]. For adolescents, psychiatric comorbidity comprises mainly mood- and anxiety disorders, obsessive-compulsive disorder, substance abuse and personality disorders [2, 4]. The results in the present study indicate that psychiatric complications might be associated with a poor outcome, which also is in line with results from previous studies [46, 47]. For example, Wentz and colleagues found that psychiatric complications might affect vulnerability for AN as well as treatment outcome [46].

There are some limitations to this study. Considerable attrition at follow-up in SwEat is one, over which we unfortunately had no control when designing the study. The amount of follow-up registrations in SwEat varies greatly between different units, probably due to varying follow-up procedures. In general, approximately 60% of initial registrations were lost to follow-up one year later [48], which might have to do with the fact that young people often wish to terminate their treatment quickly. The loss of patients at follow-up affects the generalizability of the results, although we did not find any differences of clinical relevance between followed-up and non-followed up patients in the present study. Another limitation is the missing data considering some of the variables in SwEat, due for instance to different technical issues or errors when registrations were made. As mentioned earlier, approximately 1–3% of the answers throughout the register are missing or incorrect. The fact that height and weight in some cases were self-reported by the patient might be considered a limitation, although previous results suggest that self-reported height and weight are reliable [49]. Some major limitations to this study are that we had to judge the reliability of what clinicians have reported for some of the variables and that we, due to the many years examined and the large amount of participating units, lacked control over the assessments of symptoms and diagnoses. No inter-rater agreement estimates were made and the procedure for establishing ED diagnoses varied over time as well as between units in different parts of the country. Also, the fact that some of the variables (e.g. age at first symptoms) were assessed retrospectively may have led to memory bias. These limitations, in addition to the fact that data might be affected by selection bias, are related to the naturalistic design of the study and mentioned also in previous studies as disadvantages with naturalistic register studies [12, 50]. However, the design of the study also provides several strengths. The large-scale naturalistic setting secures the generalizability to a clinical environment and offers a comparison for outcome data from treatment trials [20]. The naturalistic setting also provides a natural treatment environment for patients and clinicians, when daily routines can be followed despite study participation. An additional strength with the present study is the number of participating units, providing good national coverage.

Future research would benefit from different forms of studies focusing on how different treatment settings and approaches affect treatment outcome [51]. In recent years several different methods have been examined and tested, but nevertheless there are only a few uniform recommendations that can be applied to patients of different ages, with different diagnoses and in different social situations. The most effective treatments for adolescents with AN include family based therapy [52], but it is important to take several aspects into consideration when choosing a treatment model because of the heterogeneity of the patient group [53]. In the present study, no distinction was made between specialist and non-specialist ED units, but it has earlier been recommended that more resources should be devoted to specialist outpatient ED services with direct access from primary care for better outcome [54].

## Conclusions

Only a few naturalistic studies have focused on outcome for patients in routine care. This study shows that approximately 55% were rated by clinicians as being in remission and approximately 85% were rated as being within a healthy weight range at EOT. The results indicate that treatment for adolescents with ED in Sweden has become more effective over the past 15 years, with more patients reaching remission and a healthy weight after a shorter treatment duration. The results of the present study contribute to the scope of treatment research, but more research is needed into different forms of evidence, new research strategies and diversity of treatment approaches.

## Abbreviations

AN: Anorexia nervosa
BMI: Body mass index
DSM-IV: Diagnostic and statistical manual of mental disorders IV
ED: Eating disorder
EOT: End of treatment
SwEat: Swedish national quality register for eating disorder treatment
